# Cellular crosstalk between airway epithelial and endothelial cells regulates barrier functions during exposure to double‐stranded RNA

**DOI:** 10.1002/iid3.139

**Published:** 2017-01-18

**Authors:** Cornelia Blume, Riccardo Reale, Marie Held, Matthew Loxham, Timothy M. Millar, Jane E. Collins, Emily J. Swindle, Hywel Morgan, Donna E. Davies

**Affiliations:** ^1^ Academic Unit of Clinical and Experimental SciencesFaculty of MedicineUniversity of SouthamptonSouthamptonUnited Kingdom; ^2^ Electronics and Computer SciencesFaculty of Physical and Applied SciencesUniversity of SouthamptonSouthamptonUnited Kingdom; ^3^ Institute for Life SciencesUniversity of SouthamptonSouthamptonUnited Kingdom; ^4^ National Institute for Health ResearchSouthampton Respiratory Biomedical Research UnitUniversity Hospital SouthamptonSouthamptonUnited Kingdom

**Keywords:** Airway epithelial barrier, cellular crosstalk, endothelial barrier, fractalkine (CX_3_CL1), tumor necrosis factor alpha

## Abstract

**Introduction:**

The epithelial and endothelial barriers of the airway mucosa are critical for regulation of tissue homeostasis and protection against pathogens or other tissue damaging agents. In response to a viral infection, epithelial cells must signal to the endothelium to initiate immune cell recruitment. This is a highly temporal regulated process; however, the mechanisms of this cross‐talk are not fully understood.

**Methods:**

In a close‐contact co‐culture model of human airway epithelial and endothelial cells, cellular crosstalk was analyzed using transepithelial electrical resistance (TER) measurements, immunofluorescence, electron microscopy, and ELISA. Viral infections were simulated by exposing airway epithelial cells apically to double‐stranded RNA (Poly(I:C)). Using a microfluidic culture system, the temporal release of mediators was analyzed in the co‐culture model.

**Results:**

Within 4 h of challenge, double‐stranded RNA induced the release of TNF‐α by epithelial cells. This activated endothelial cells by triggering the release of the chemoattractant CX_3_CL1 (fractalkine) by 8 h post‐challenge and expression of adhesion molecules E‐selectin and ICAM‐1. These responses were significantly reduced by neutralising TNF‐α.

**Conclusion:**

By facilitating kinetic profiling, the microfluidic co‐culture system has enabled identification of a key signaling mechanism between the epithelial and endothelial barriers. Better understanding of cell–cell cross‐talk and its regulatory mechanisms has the potential to identify new therapeutic strategies to control airway inflammation.

## Introduction

With an estimated surface area of around 140 m^2^, the lung is the organ with the largest interface with the external environment 1. During inspiration, the lung epithelial surface is exposed to a variety of naturally occurring and anthropogenic substances with potential to do harm. However, the filtering and innate protective mechanisms of the airways inactivate and/or remove most of these substances without any need for immune cell activation 2. As well as contributing to tissue homeostasis through its barrier functions, the airway epithelium must be able to respond appropriately when it is compromised by signaling to cells of the innate and adaptive immune system 3. These immune cells may reside locally, or be recruited from the circulation via endothelial cell activation. Although the mechanisms of epithelial–endothelial crosstalk are not fully understood, communication can be achieved through release of a variety of mediators including cytokines, chemokines, growth factors, lipids, and other small molecules such as reactive oxygen species (ROS) or nitric oxide (NO) 2. The integrated responses arising from this cell–cell communication can be observed readily in animal models *in vivo*, however *in vitro* models using human cells are more amenable for dissection of mechanisms of cell–cell communication and identification of key cell‐type specific mediators with relevance to human disease 4.

Traditionally, cellular crosstalk has be analyzed *in vitro* using conditioned media from one cell type to stimulate second cell type. For example, by using conditioned media from endothelial cells, it has been shown that lung endothelial cells improve the physical barrier properties of alveolar epithelial cells, while factors from brain‐derived endothelial cells diminish the epithelial barrier 5. However, use of conditioned media overlooks the close spatial relationship between individual cell types within a tissue, especially direct cell–cell contacts. Crucially, it neglects the temporal evolution of mediator release. Consequently, co‐culture models of different cell types have been developed to reflect the *in vivo* situation more closely. In most cases, the cell types were separated by a permeable filter support, with one cell type cultured in the apical and the other in the basolateral compartment. For example, *in vitro* models of the air–blood–barrier consisting of lung epithelial and endothelial cells have been used to study the mechanisms of acute lung injury 6. Air–blood–barrier models have also been used to analyze the passage of nanoparticles across the barrier and to evaluate their immune–modulatory capacity 7, 8, 9. These improved models have led to the proposal that airway epithelial–endothelial co‐culture models have the potential to replace *in vivo* animal studies for analysis of pulmonary toxicity 10.

Although commonly used co‐culture models represent an advance, these models lack the constant exchange of metabolites or diffusion of mediators by the circulation as observed *in vivo*. To address this problem, we have developed a dynamic microfluidic culture system which mimics interstitial flow, enabling supply of nutrients, removal of metabolites, and analysis of time‐dependent mediator release with much higher sensitivity 11. In the current study, we have exploited this system to analyze the temporal crosstalk between lung epithelial and endothelial cellular barriers in response to respiratory viral infections. We hypothesized that the epithelium senses the infections and activates endothelial cells to facilitate immune cell infiltration. We utilized a microfluidic culture system comprising of a close‐contact epithelial–endothelial co‐culture model exposed to double stranded RNA, a virus‐associated molecular pattern. We found that epithelial‐derived TNF‐α activated endothelial cells to release CX_3_CL1, a chemotactic molecule for monocytes, NK cells, and CD4^+^ T lymphocytes 12, 13. The analysis of cellular crosstalk and the kinetics of mediator release are important in order to understand the regulation of tissue homeostasis. This approach provides insight into the underlying mechanisms of cellular pathology in diseases including asthma and chronic obstructive pulmonary disease thereby helping to identify therapeutic targets.

## Materials and Methods

### Cell culture

All procedures for the collection of human umbilical cords and isolation of human umbilical vein endothelial cells (HUVECs) were approved by the Southampton and South West Hampshire Research Ethics Committee (REC Ref: 07/H0502/83). HUVECs were isolated from human umbilical cords as previously described 14. Briefly, the veins of umbilical cords were incubated with Type I collagenase solution (1 mg/ml) for 10 min to remove endothelial cells. Cells in solution were centrifuged and cultured on gelatin‐coated tissue culture flasks in endothelial culture medium (M199 medium supplemented with L‐Glutamine, penicillin/streptomycin (Life Technologies, Paisley, UK) and 20% human serum) until ∼80% confluent. Experiments were performed with endothelial cells in passage 1.

The human bronchial epithelial cell line, 16HBE14o‐ (a gift from Prof. D.C. Gruenert, San Francisco, USA), was maintained in epithelial medium (minimum essential medium (MEM) with Glutamax and supplemented with 10% foetal bovine serum and penicillin/streptomycin (Life Technologies)). Cell culture flasks were coated with PureCol collagen I (Advanced BioMatrix, San Diego, CA). All experiments were performed with epithelial cells for no more than 20 passages in culture.

### Epithelial–endothelial co‐culture

Transwell® polyester membrane cell culture inserts (6.5 mm diameter, 0.4 μm pore size; Corning Life Sciences, Amsterdam, The Netherlands) were used for culturing human airway epithelial and endothelial cells. Airway epithelial cells were cultured on the apical side and endothelial cells on the basal side of the permeable culture insert. After coating both sides of the membrane with collagen, inserts were turned upside‐down and 5 × 10^4^ HUVECs in a volume of 50 μl endothelial medium were seeded on the basal side of the membrane. Endothelial cells were left in a humidified incubator at 37°C, 5% CO_2_ for 2 h to adhere. Non‐adherent cells were gently washed off and the inserts replaced into a 24‐well plate with 500 μl endothelial medium in the basolateral compartment. Airway epithelial cells were seeded in the apical compartment at a density of 1.5 × 10^5^ cells in 200 μl epithelial cell medium. Cells were co‐cultured for up to 8 days and media was changed every 2 and 3 days. The formation of the physical barrier was monitored by measuring the ionic permeability by transepithelial resistance (TER) using an EVOM voltohmmeter (World Precision Instruments, Aston, UK). TER measurements were corrected for the resistance of an empty Transwell (170 Ω) and were expressed as Ω × cm^2^.

### Transmission electron microscopy

Cell culture inserts were fixed with 3% glutaraldehyde and 4% paraformaldehyde in 0.1 M PIPES buffer and contrast stained with 1% osmium tetroxide and 2% uranyl acetate in 0.1 M PIPES buffer. After dehydration in ethanol and acetonitrile, the membranes were embedded in Spurr resin. Ultrathin sections (∼60 nm) were stained with Reynolds’ lead citrate stain and analyzed with a H7000 transmission electron microscope (Hitachi High‐Technologies Europe GmbH, Maidenhead, UK).

### Immunofluorescence staining

For immunofluorescence staining, cells were fixed in 4% paraformaldehyde, permeabilized with 0.1% Triton X‐100 and blocked with 1% BSA in PBS. Membranes were cut from the inserts and epithelial cells were stained with a mouse anti‐human occludin‐AlexaFluor488 (clone OC‐3F10, Life Technologies) and Acti‐stain 555 phalloidin‐(Cytoskeleton Inc., Denver, CO). Endothelial cells were stained with mouse anti‐human ICAM‐1 and E‐selectin monoclonal antibodies (clone BBIG‐I1 and BBIG‐E1 respectively, R&D Systems, Abingdon, UK) and AlexaFluor®488 conjugated goat anti‐mouse IgG1 secondary antibody (Life Technologies). Actin filaments were stained using Acti‐stain 555 phalloidin. Stained membranes were mounted on slides using ProLong Gold antifade reagent with DAPI (Life Technologies) and analyzed with a LSM6000 microscope (Leica Microsystems, Wetzlar, Germany). z‐Stacks were deconvoluted using Leica Application Suite software (Leica Microsystems) and z‐projections and orthogonal views were performed using ImageJ software (https://imagej.nih.gov/ij/index.html).

### Microfluidic culture system

**Figure 1:**
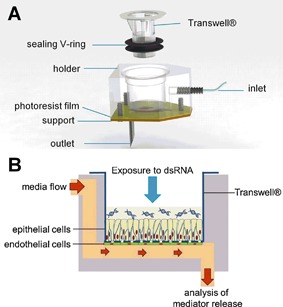
Design of the microfluidic culture system. A: A photoresist dry film forming the microfluidic channel is laminated onto a support layer on which a holder is fixed. A permeable Transwell® support is inserted and sealed by a V‐ring. B: Schematic section of a epithelial–endothelial co‐culture under microfluidic culture conditions.

The design, fabrication, and validation of the microfluidic culture system has been described in detail previously 11. After 6 days in co‐culture, cells on inserts were transferred to microfluidic culture device and perfused with endothelial medium at a flow rate of 30 μl/h (Fig. 1). Following an equilibration phase of 1h, epithelial cells were apically exposed to 5 μg/ml Poly(I:C) (HMW, 1.5–8 kb, Invivogen, Toulouse, France) to mimic a viral infection. Basolateral secretions were collected with an automated fraction collector every 2 h for a period of 24 h. Control experiments were performed under static culture conditions in 24‐well plates with 200 μl apical and 500 μl basolateral medium.

### Release of mediators

The release of TNF‐α and CX_3_CL1 (fractalkine) into the basolateral medium was analyzed by ELISA using Human TNF‐α DuoSet and Human CX_3_CL1/Fractalkine DuoSet kits (R&D Systems). For blocking experiments, the soluble TNF‐α receptor fusion protein, Enbrel^®^ (Entanercept) (Wyeth Europa Ltd., Maidenhead, UK) was added to the apical and basolateral medium at a concentration of 10 μg/ml, 1 h prior stimulation.

### Macromolecular permeability

FITC‐labeled dextran with an average molecular weight of 4 kDa was added to the apical medium at a concentration of 2 mg/ml for the last 3 h of stimulation. The amount of FITC‐dextran passage to the basolateral compartment was analyzed by measuring the fluorescence intensity and taken as a measure of macromolecular permeability.

### Statistical analysis

Statistical evaluation was performed using the software SigmaPlot 12.5 (Systat Software Inc., London, UK). If not stated otherwise, related samples were analyzed for statistical significance using the non‐parametric Wilcoxon test. Differences were regarded as significant when *P* ≤ 0.05.

## Results

### Improved physical barrier in co‐culture

**Figure 2:**
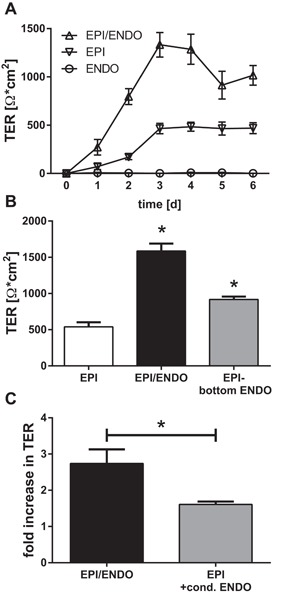
Enhanced barrier properties in epithelial–endothelial co‐cultures. The integrity of the epithelial and endothelial barriers was monitored by measuring the transepithelial (or endothelial) resistance (TER). A: Epithelial cells (EPI) were cultured on the apical and endothelial cells (ENDO) on the basolateral side of permeable filter supports for up to 6 days (Mean ± SEM, *n* = 11–16 independent experiments). B: Comparison of TER measurements on day 3 of epithelial mono‐cultures (clear bar), epithelial–endothelial co‐culture in close proximity on filter supports (black bar) and co‐cultures with endothelial cells grown on the bottom of the well at a distance to epithelial cells (gray bar) (Mean ± SEM; *n* = 7 independent experiments; *P ≤ 0.05 compared to EPI (Wilcoxon)). C: Effect of endothelial cell conditioned medium on TER of epithelial cells after 3 days of culture. TER was normalized to the TER of epithelial monocultures (Mean ± SEM; *n* = 10 independent experiments; *P ≤ 0.05 (Wilcoxon)).

The barrier properties of the co‐culture model were monitored by transepithelial resistance (TER), a measure of the ionic permeability. As shown in Figure 2A, the TER in the co‐cultures was significantly increased from day 1 of culture compared to epithelial mono‐cultures. At day 3 of culture, a maximum was reached with the co‐cultures showing more than a twofold increase in TER compared with epithelial mono‐cultures. Over the following days, the TER was slightly reduced in the co‐cultures, but remained around twofold higher than the epithelial mono‐cultures. The close proximity of the epithelial and endothelial cells in the co‐culture model was important for the observed decrease in ionic permeability in the co‐cultures, since culturing the endothelial cells at the bottom of the culture plate rather than directly on the basolateral side of the permeable filter support resulted in a smaller increase in TER (Fig. 2B). Furthermore, soluble factors released by endothelial cells triggered the increase in epithelial TER, since conditioned media from endothelial cells caused a 1.5‐fold increase in TER (Fig. 2C). However, this increase caused by conditioned media was lower than the increase in TER observed in the co‐culture model again confirming the importance of the close proximity of the two cell types.

### Morphology of co‐cultures

**Figure 3:**
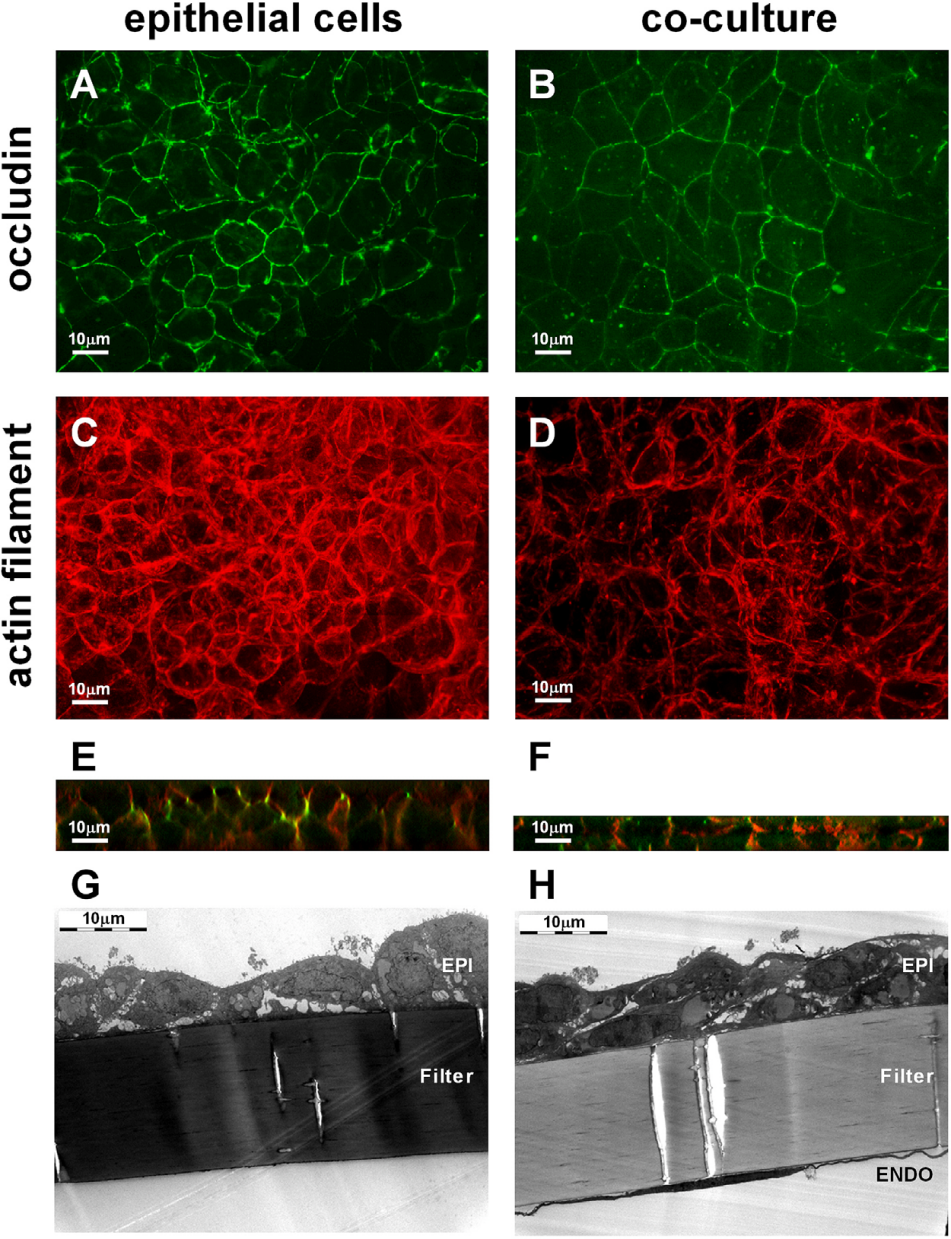
Epithelial cells change the morphology during co‐culture with endothelial cells. Epithelial (EPI) and endothelial cells (ENDO) were co‐cultured for 6–8 days (right panels) and the morphology of the epithelial cells compared to epithelial monocultures (left panels) by microscopy. Analysis by fluorescence microscopy was performed using an anti‐occludin antibody (green, panels A and B) and an actin filament specific dye (red, panels C and D). A z‐projection of the epithelial cell layer is shown. E and F: Orthogonal views of the z‐stacks. G and H: Electron scanning microscopy images of epithelial monoculture (G) and epithelial–endothelial co‐culture (H). Images are representative of three independent experiments.

Epithelial and endothelial cells were cultured together in close proximity for a period in which the polarization of the epithelial layer occurred. After 6–8 days in culture, immunofluorescence microscopy showed that epithelial cells in either mono‐ or co‐culture had zonular apicolateral staining at cell–cell contacts with antibodies specific for the tight junction protein, occludin, with F‐actin showing a broadly similar organization (Fig. 3A–D). However, in the co‐cultures the occludin was more regularly organized at the subapical lateral regions of the cells (Fig. 3E and F) and the thickness of the epithelial cell sheet (10.82 μm ± 0.83 mean ± SD) was significantly reduced compared with monocultures (21.15 μm ± 3.34 mean ± SD; *P* = 0.0214; paired *t*‐test). Electron microscopy confirmed that the epithelial cells in the co‐cultures had formed a more even cell sheet with a pseudostratified structure whereas the epithelial monocultures showed little evidence of stratification and the apical surface was more irregular (Fig. 3G and H).

### Effect of poly(I:C) on physical barrier

**Figure 4:**
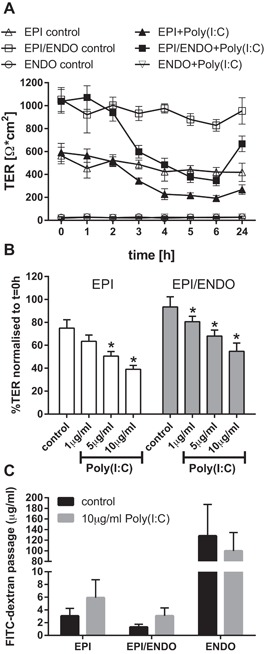
Effect of Poly(I:C) on the physical barrier properties. Epithelial (EPI) and endothelial cells (ENDO) were co‐cultured for 6 days and subsequently exposed apically with Poly(I:C), a mimic of viral double‐stranded RNA. Epithelial and endothelial monocultures were used as controls. A: Time‐dependent changes of the ionic permeability measured by transepithelial resistance (TER) after stimulation with 5 μg/ml Poly(I:C) (Mean ± SEM; *n* = 8 independent experiments). B: Dose‐dependent effect of Poly(I:C) on the TER of epithelial monocultures and co‐cultures at 24 h. The TER at *t* = 24 h is normalized to the TER at *t* = 0 h (Mean ± SEM, *n* = 8 independent experiments; *P ≤ 0.05 compared to untreated control (Wilcoxon)). C: Macro‐molecular permeability of the barrier after Poly(I:C) stimulation. Mono‐ or co‐cultures were apically exposed with FITC‐dextran after 21 h of Poly(I:C) stimulation and the passage of FITC‐dextran into the basolateral compartment was calculated by measuring the fluorescence intensity after 3 h (Mean ± SEM, *n* = 5 independent experiments).

As previously reported 15, apical exposure of polarized epithelial monocultures to double‐stranded RNA (Poly(I:C)), a virus‐associated molecular pattern that mimics viral infections, caused a reduction in the TER as early as 3 h after exposure (Fig. 4A). The minimum occurred 6 h after exposure with a level that was around 50% lower than the untreated control, and there was a slight recovery by 24 h after exposure. In the co‐culture, a similar response to Poly(I:C) was detected with a drop in TER 3 h after exposure and a 50% decrease compared with the untreated control. However, the absolute TER value of the co‐cultures with Poly(I:C) treatment remained higher and was at the same level of the untreated epithelial monoculture. Both epithelial mono‐cultures and epithelial–endothelial co‐cultures showed a dose‐dependent reduction in TER after stimulation with Poly(I:C) for 24 h (Fig. 4B). Although Poly(I:C) caused a significant increase in ionic permeability, this was associated with only small increases in macromolecular permeability as determined by passage of FITC‐dextran (Fig. 4C), but again the overall permeability of the co‐cultures was lower and was similar to the untreated epithelial monoculture. In contrast to cultures containing epithelial cells, the endothelial cell monocultures had comparatively low TER values (25 Ω × cm^2^) compared to epithelial cells (564.7 Ω × cm^2^ ± 72.6 SEM) and epithelial–endothelial co‐cultures (1053.9 Ω × cm^2^ ± 104.7 SEM). Additionally, endothelial cells exhibited 40–50 times higher macromolecular permeability, suggesting that the epithelial cells contributed most to the tight barrier properties of the co‐culture.

Using static culture conditions, we analyzed mediator release in response to Poly(I:C) challenge (Supplementary Fig. S1). TNF‐α release was significantly increased by Poly(I:C) in epithelial monocultures and co‐cultures, while endothelial monocultures released very low levels of TNF‐α which were reduced after Poly(I:C) treatment (Supplementary Fig. S1A). Poly(I:C) not only stimulated release of CX_3_CL1 in the co‐culture (Supplementary Fig. S1B), but also directly stimulated CX_3_CL1 release from endothelial cells. Interestingly, the baseline levels of released CX_3_CL1 were increased in the co‐culture, an effect that might be caused by soluble factors released by epithelial cells. Taken together, our data suggested that Poly(I:C) drives epithelial release of TNF‐α and endothelial release of CX_3_CL1.

### Kinetic of mediator release

**Figure 5:**
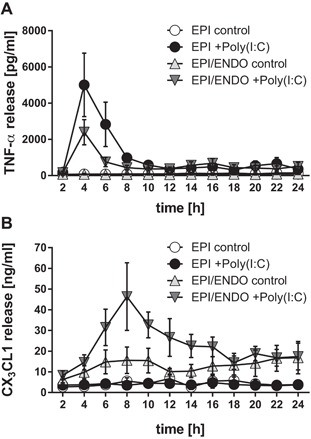
Time‐dependent release of mediators after Poly(I:C) stimulation. After 6 days in culture, epithelial monocultures or co‐cultures were transferred to the microfluidic culture system and apically stimulated with 5 μg/ml Poly(I:C). The basolateral flow through was collected in 2 h intervals with an automated fraction collector and mediator release analyzed by ELISA. A: Basolateral release of TNF‐α in response to Poly(I:C). B: Time‐dependent release of CX_3_CL1 (fractalkine). Mean ± SEM, *n* = 7–8 independent experiments.

Using a microfluidic culture system (Fig. 1), we were able to analyze the kinetics of mediator release in co‐cultures and epithelial mono‐cultures. As shown in Figure 5A, Poly(I:C) induced release of TNF‐α which peaked as early as 4 h and was reduced to background level 8 h after stimulation. The kinetics of TNF‐α release was comparable in epithelial monocultures and co‐cultures, however, consistent with the static culture system, release of TNF‐α in the co‐culture was slightly lower than in epithelial monocultures. This might be explained by utilization of TNF‐α binding to receptors present on endothelial cells. Similar to the static cultures, CX_3_CL1 release was only detected in the co‐cultures and not in epithelial monocultures, suggesting that the CX_3_CL1 release is derived from the endothelial cells (Fig. 5B). In the co‐cultures, maximal CX_3_CL1 release occurred 8 h after stimulation and returned to baseline level by 18 h.

### Regulation of endothelial CX_3_CL1 release

**Figure 6:**
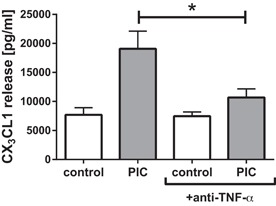
Anti‐TNF‐α attenuates the Poly(I:C) induced release of CX_3_CL1 (fractalkine) in the co‐cultures. Co‐cultures under static conditions were pre‐incubated for 1 h with anti‐TNF‐α and subsequently stimulated with Poly(I:C) apically. Basolateral release of CX_3_CL1 (fractalkine) was analyzed after 24 h by ELISA. Mean ± SEM, *n* = 5 independent experiments; *P ≤ 0.05 (one‐tailed paired *t*‐test).

Although Poly(I:C) was able to directly stimulate CX_3_CL1 release by endothelial cells, the changes in macromolecular permeability of the epithelial–endothelial cell co‐cultures were low, suggesting that little Poly(I:C) would penetrate across the epithelial barrier and come into direct contact with the endothelial cells. However, the timing of cytokine release detected using the microfluidic culture system suggested that the epithelial‐derived TNF‐α observed 4 h after stimulation might trigger endothelial CX_3_CL1 release observed 8 h after stimulation. To test this hypothesis, we used Etanercept, a soluble TNF‐α receptor to antagonize the effect of TNF‐α. This caused a significant reduction of CX_3_CL1 release by the co‐cultures after Poly(I:C) stimulation (Fig. 6), indicating that cellular cross talk is occurring with epithelial‐derived TNF‐α driving the release of endothelial CX_3_CL1, rather than the Poly(I:C) directly stimulating the endothelial cells. In contrast, blocking TNF‐α did not alter the Poly(I:C)‐induced effect on TER (Supplementary Fig. S2A and B).

### Expression of endothelial adhesion molecules

**Figure 7:**
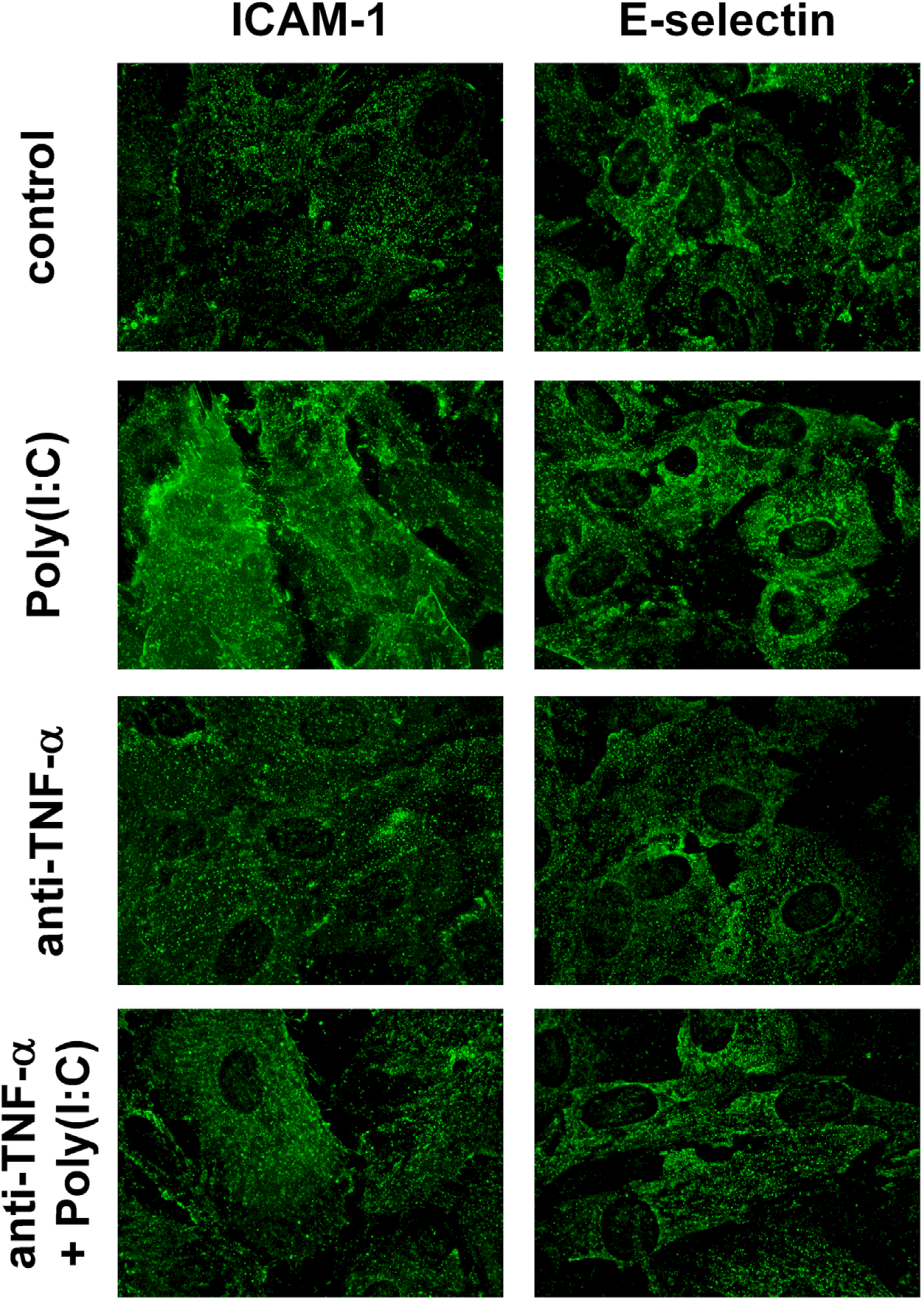
Poly(I:C) induced expression of endothelial adhesion molecules is inhibited by anti‐TNF‐α treatment in the co‐cultures. Co‐cultures were pre‐treated with anti‐TNF‐α for 1 h cultures and subsequently stimulated with 5 μg/ml Poly(I:C) apically. Expression of ICAM‐1 (left panels) and E‐selectin (right panels) was analyzed by fluorescence microscopy after 24 h. Images are representative of three independent experiments.

After Poly(I:C) stimulation, the expression of endothelial adhesion molecules in epithelial–endothelial co‐cultures was analyzed by fluorescence microscopy. As shown in Figure 7, the endothelial cells showed an increased expression of the adhesion molecules, ICAM‐1 and E‐selectin after Poly(I:C) stimulation of the co‐cultures. This increase in expression was reduced by neutralizing TNF‐α, indicating that epithelial‐derived TNF‐α also triggered the expression of endothelial adhesion molecules in response to double‐stranded RNA.

## Discussion

In this study, we demonstrated that cellular crosstalk plays an important role in co‐ordinating barrier functions of the airway epithelium and endothelium. Through use of a close contact co‐culture model, we showed that the epithelial barrier properties are enhanced, an effect that is mediated by the presence of endothelial‐derived mediators. In conjunction, we observed morphological changes of the epithelium suggesting that endothelial cells support the maturation of the epithelium into a pseudostratified layer, although complete differentiation into a mucociliary epithelium was not observed. Furthermore, in response to double‐stranded RNA (Poly(I:C)), the epithelium responds rapidly and transiently by releasing TNF‐α which activates endothelial cells to produce CX_3_CL1 and express adhesion molecules. This co‐ordinated response is important in regulating immune cell transmigration across the endothelial barrier and into the tissue. Although microfluidic models of the alveolar‐capillary interface have been developed 16, 17, our microfluidic culture model of the airways allowed us to analyze for the first time the temporal release of these mediators in response to double‐stranded RNA and to show that TNF‐α release by epithelial cells is preceded by, and required for, CX_3_CL1 release by endothelial cells. A key advantage of our microfluidic culture system over conventional static culture is that it simulates interstitial flow and limits acculmulation of mediators, as occurs *in vivo*. Thus, it allows for temporal control of mediator release to be analyzed in great detail, at shorter time intervals, and with a higher sensitivity. This will allow detailed investigations of the primary or up‐stream regulatory mechanisms that control cellular crosstalk and help to identify key processes that are dysfunctional in diseases, especially inflammatory chronic lung diseases like asthma and chronic obstructive pulmonary disease (COPD). Furthermore, this complex dynamic human 3D *in vitro* model has the potential to reduce and replace animal experiments for analysing pathological mechanisms underlying chronic lung diseases, as animal models have only limited transferability into the human disease 18.

Improvement of the physical barrier properties during lung epithelial–endothelial co‐cultures has been shown previously. For example, Chowdhury et al. 19 reported improved physical barrier properties measured by TER in an airway epithelial–endothelial co‐culture model, an effect that was mediated by endothelial‐derived factors. However, they did not observe morphological changes in the airway epithelium in the co‐cultures. This might be due to a difference in the co‐culture model, since the endothelial cells were introduced after completion of epithelial polarization while in the current study, epithelial and endothelial cells were co‐cultured during the period of epithelial polarization. Interestingly, this barrier improving effect of endothelial cells seems to be tissue specific, since endothelial cells derived from brain tissue have been reported to cause a weakening of the physical barrier of lung epithelial cells 5. Reduced barrier properties have also been reported in retinal epithelial and endothelial co‐cultures 20. The endothelial‐derived factors mediating the enhancing or reducing effects on the epithelial barrier and the mechanisms are still unknown.

Poly(I:C), an analog of double‐stranded RNA that mimics viral replication, has been evaluated for its effects on airway barrier functions previously. Airway epithelial cells express various pattern‐recognition receptors (PRRs) to sense double‐stranded RNA, including toll‐like receptor 3 (TLR3), protein kinase D (PKD), and cytoplasmic helicases like RIG‐I and MDA5 15, 21. Similar to our data, increased ionic permeability has been detected within the first 3 h after exposure of polarized 16HBE14o‐ cells to Poly(I:C) and this was linked to an increase in macromolecular permeability 15. The decrease in physical barrier integrity has been associated with disassembly of adherens and tight junction proteins, a process that is thought to be mediated by PKD 15. However, although Poly(I:C) increased the ionic permeability of the co‐culture, the effect on macromolecular permeability was small and the permeability was similar to that of untreated epithelial mono‐cultures which exhibit good barrier properties and low macromolecular permeability. Consequently, it seemed unlikely that apically applied Poly(I:C) would be able to penetrate the epithelial barrier to directly activate the underlying endothelial cells.

Double‐stranded RNA is also able to induce the release of inflammatory mediators by airway epithelial cells, which include the release of CXCL8/IL‐8, CXCL10/IP‐10, IFN‐β, and TNF‐α 22. Here, we show that double‐stranded RNA induced the release of TNF‐α by airway epithelial cells. TNF‐α is an important inflammatory mediator that acts on many cell types including fibroblasts, endothelial cells, and immune cells as well as epithelial cells themselves. For example, exposure of differentiated bronchial epithelial cells to TNF‐α over 4 days resulted in an increased ionic and macromolecular permeability of the barrier and stimulated release of cytokines and metalloproteases 23. TNF‐α is also thought to trigger mucus production in airway epithelial cells 24. However, *in vivo*, release of mediators shows a time‐dependency that facilitates cellular crosstalk in response to environmental impacts. Using a dynamic microfluidic *in vitro* culture system that simulates interstitial flow, we have been able to analyze the kinetics of the mediator release with a higher sensitivity and accuracy compared to conventional static culture conditions 11. By using this dynamic culture system in combination with the epithelial–endothelial co‐culture model, we were able to show that epithelial cells release TNF‐α rapidly after challenge with Poly(I:C), peaking 4 h after exposure and falling to basal levels by 10–12 h. Although we showed that endothelial cells can respond to direct stimulation by Poly(I:C), in the co‐culture system with both epithelial and endothelial barriers acting in a co‐ordinated fashion, early release of TNF‐α by airway epithelial cells was shown to be responsible for endothelial cell release of CX_3_CL1. Epithelial‐derived TNF‐α also induced the expression of the adhesion molecules ICAM‐1 and E‐selectin.

TNF‐α is a well‐known regulator of endothelial functions and its mechanisms were studied extensively 25, 26. Using endothelial mono‐cultures, induction of CX_3_CL1 after stimulation with exogenous TNF‐α has been shown previously 27, 28. By facilitating kinetic profiling, the microfluidic co‐culture system has enabled identification of TNF‐α as a key endogenous signaling mechanism between the epithelial and endothelial barriers. It is thought that TNF‐α induces the transcription of CX_3_CL1 by a phosphatidylinositol 3′‐kinase and NF‐κB mediated pathway 27. Additionally, TNF‐α has been shown to stabilize CX_3_CL1 mRNA via a p38 MAPK‐dependent mechanism on a post‐transcriptional level, which results in synergistically induced CX_3_CL1 expression in HUVECs after TNF‐α and IFN‐γ stimulation 28. Released soluble CX_3_CL1 is involved in leukocyte trafficking by attracting and activating CD8+ and CD4+ T cells, natural killer cells, dendritic cells, and monocytes 29. There is evidence that CX_3_CL1 is linked to inflammatory chronic lung diseases as raised levels of CX_3_CL1 have been shown in asthma and COPD 30, 31.

Activation of endothelial cells by TNF‐α also results in the expression of adhesion molecules facilitating the transmigration of leukocytes across the endothelial barrier. For example, TNF‐α is a well‐known inducer of endothelial adhesion molecule expression like E‐selectin and ICAM‐1 which is mediated by the NF‐κB and AP‐1 signaling pathways 25, 26. Recent data showed that the expression of E‐selectin is induced by TNF‐α by a p66^Shc^ and JNK‐mediated pathway and results in increased transmigration of leukocytes 32. As blockade of TNF‐α only partially prevents endothelial activation, other epithelial‐derived factors, such as IL‐8 or IFN‐β, may contribute to endothelial activation. IL‐8 has been shown to initiate the reorganization of the endothelial cytoskeleton 33 and IFN‐β modulates endothelial expression of ICAM‐1 and MHC class I and II molecules 34.

After endothelial activation the recruitment of immune cells is mediated by a highly regulated adhesion cascade including the capture, rolling, crawling, and finally the transmigration of the immune cell across the endothelial barrier, a process that has been intensively studied 35, 36. As TNF‐α plays a central role in the regulation of inflammation, TNF‐blocking biologicals are already approved for the treatment of inflammatory diseases including rheumatoid arthritis, inflammatory bowel disease, and psoriasis 37, 38. The efficacy of anti‐TNF‐α drugs in pulmonary inflammatory diseases such as asthma has been investigated 39, 40, 41, as well as the use of other non‐biological drugs like polyphenols 42. However, the role of TNF‐α mediated endothelial activation in virally induced lung inflammation is less well characterized. As viral infections are the most common trigger of exacerbations in chronic lung diseases like asthma and COPD, targeting adhesion molecules involved in immune cell recruitment is a promising therapeutic strategy 43. Using an epithelial–endothelial co‐culture model in combination with a dynamic culture platform allows us to identify a key signaling mechanism between the epithelial and endothelial barriers. This should enhance our understanding of the regulatory mechanisms during airway inflammation and contribute to the identification of new, more effective drugs targeting chronic airway inflammation. Additionally, the dynamic co‐culture model of the airway mucosa is an ideal tool for testing candidate drugs for their efficacy in the pre‐clinical phase, since it incorporates the aspect of cellular crosstalk and the dynamic flow of metabolites observed *in vivo*.

### Limitations of the study

The aim of this study was to highlight the potential for utilization of microfluidic culture systems with epithelial–endothelial co‐culture models to enable kinetic analysis of the mediators involved in cell–cell communication between the epithelial and endothelial barriers. In order to facilitate uptake of the model by the scientific community and to ensure accessibility to human cell material, we utilized an airway epithelial cell line (16HBE) that forms a polarized barrier in culture and human umbilical cord endothelial cells (HUVECs). Both cell types have been used extensively to study airway epithelial and endothelial functions. For example, we and others have found that 16HBE cells and fully differentiated primary bronchial epithelial cells respond similar to challenge with pollen 44 or Poly(I:C) 45. However, in future work it will be important to evaluate the co‐culture model using fully differentiated primary bronchial epithelial cells and pulmonary (or bronchial) microvascular endothelial cells. Furthermore, while we elected to use Poly(I:C) as a proto‐typical pathogen‐associated molecular pattern (PAMP) that mimics the production of double‐stranded RNA during viral replication, this only reflects one aspect of viral infection. In order to reflect the complexity of human rhinovirus infections, further work would be required using infectious respiratory viruses.

## Author contributions

C.B., R.R., and M.H. designed and performed the experiments; M.L. assisted with electron microscopy analysis; E.J.S, H.M., D.E.D., J.E.C., and T.M.M. conceived the study; C.B. and D.E.D analyzed the data and prepared the manuscript. All authors contributed to the discussions of the project and reviewed the manuscript.

### Conflict of interest

No competing interests declared.

## Supporting information

Additional supporting information may be found in the online version of this article at the publisher's web‐site.

**Figure S1**. Poly(I:C) induces the release of TNF‐α by epithelial cells and fractalkine (CX_3_CL1) by endothelial cells. After 6 days in culture, mono‐ or co‐cultures were apically stimulated with 5 μg/ml Poly(I:C) for 24 h and the release of TNF‐α (A) or CX_3_CL1 (fractalkine) (B) into the basolateral compartment analyzed by ELISA. Mean ± SEM; *n *= 7–9 independent experiments; *P ≤ 0.05 compared to untreated control (Wilcoxon).
**Figure S2**. Anti‐TNF‐α treatment does not change the physical barrier properties in epithelial mono‐ and co‐cultures. After pre‐treatment with anti‐TNF‐α for 1 h cultures were apically stimulated with 5 μg/ml Poly(I:C) and the transepithelial resistance (TER) measured over time. A: Epithelial monocultures; B: epithelial‐endothelial co‐cultures. Mean ±** **SEM, *n* = 3 independent experiments.
