# Supplementary material for: Cellular crosstalk between airway epithelial and endothelial cells regulates barrier functions during exposure to double‐stranded RNA
Source: Immun Inflamm Dis. 2017 Jan 18;5(1):45–56. doi: 10.1002/iid3.139 (PMC5322162; doi:10.1002/iid3.139)
Supplement: Supplementary file 1 — Figure S1. Poly(I:C) induces the release of TNF‐α by epithelial cells and fractalkine (CX3CL1) by endothelial cells. After 6 days in culture, mono‐ or co‐cultures were apically stimulated with 5 μg/ml Poly(I:C) for 24 h and the release of TNF‐α (A) or CX3CL1 (fractalkine) (B) into the basolateral compartment analyzed by ELISA. Mean ± SEM; n = 7–9 independent experiments; *P ≤ 0.05 compared to untreated control (Wilcoxon). Figure S2. Anti‐TNF‐α treatment does not change the physical barrier properties in epithelial mono‐ and co‐cultures. After pre‐treatment with anti‐TNF‐α for 1 h cultures were apically stimulated with 5 μg/ml Poly(I:C) and the transepithelial resistance (TER) measured over time. A: Epithelial monocultures; B: epithelial‐endothelial co‐cultures. Mean ± SEM, n = 3 independent experiments. [file IID3-5-45-s001.pdf]

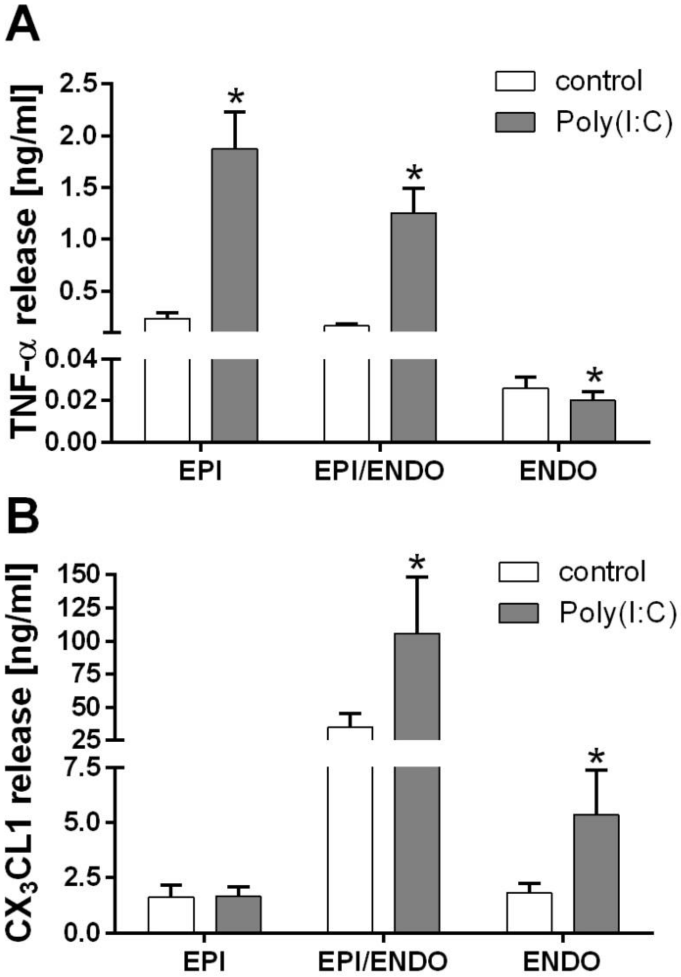

Fig. S1: Poly(I:C) induces the release of TNF-α by epithelial cells and fractalkine (CX_3CL1) by endothelial cells. After 6 days in culture, mono- or co-cultures were apically stimulated with 5µg/ml Poly(I:C) for 24h and the release of TNF-α (A) or CX_3CL1 (fractalkine) (B) into the basolateral compartment analysed by ELISA. Mean±SEM; n=7-9 independent experiments; *: $p \leq 0.05$ compared to untreated control (Wilcoxon).

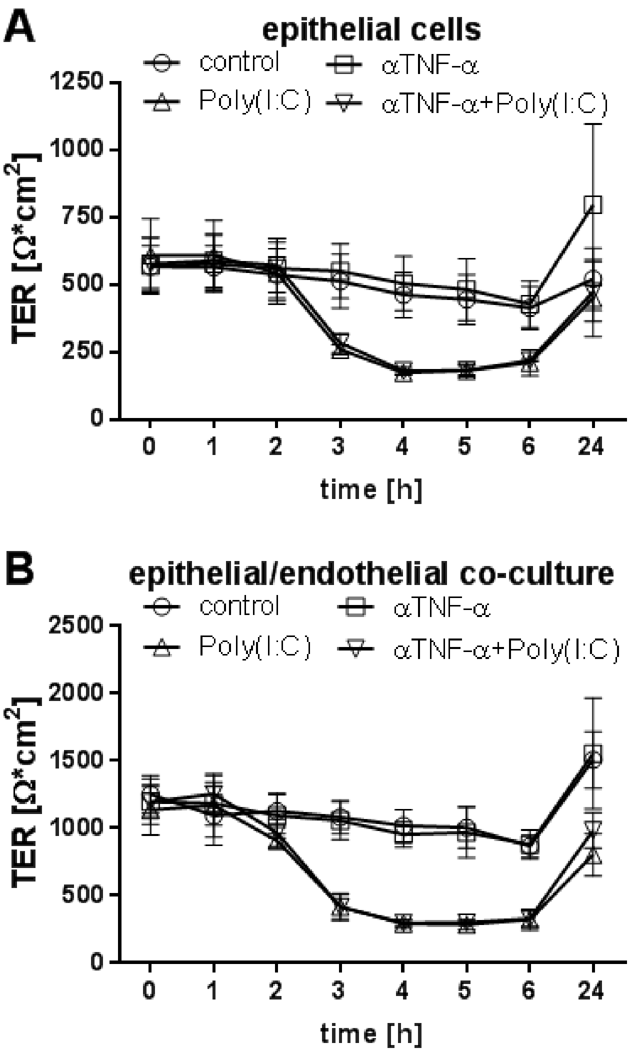

Fig. S2: Anti-TNF-α treatment does not change the physical barrier properties in epithelial mono-and co-cultures. After pre-treatment with anti-TNF-α for 1h cultures were apically stimulated with 5µg/ml Poly(I:C) and the transepithelial resistance (TER) measured over time. A: Epithelial monocultures; B: epithelial-endothelial co-cultures. Mean±SEM, n=3 independent experiments.
